# The Plight of the Metabolite: Oxidative Stress and Tear Film Destabilisation Evident in Ocular Allergy Sufferers across Seasons in Victoria, Australia

**DOI:** 10.3390/ijms25074019

**Published:** 2024-04-04

**Authors:** Esrin Aydin, Damien L. Callahan, Luke Chong, Serap Azizoglu, Moneisha Gokhale, Cenk Suphioglu

**Affiliations:** 1 NeuroAllergy Research Lab (NARL), School of Life and Environmental Sciences, Deakin University, Geelong 3217, Australia; eaydi@deakin.edu.au; 2 School of Medicine, Deakin University, Waurn Ponds 3216, Australia; luke.chong@deakin.edu.au (L.C.); serap.azizoglu@deakin.edu.au (S.A.); moneisha.gokhale@deakin.edu.au (M.G.); 3 School of Life and Environmental Sciences, Deakin University, Burwood 3125, Australia; damien.callahan@deakin.edu.au

**Keywords:** immunometabolism, human tears, metabolomics, lipidomics, mass spectrometry, tear film, oxidative stress, inflammation, ocular allergy

## Abstract

Ocular allergy (OA) is characterised by ocular surface itchiness, redness, and inflammation in response to allergen exposure. The primary aim of this study was to assess differences in the human tear metabolome and lipidome between OA and healthy controls (HCs) across peak allergy (spring–summer) and off-peak (autumn–winter) seasons in Victoria, Australia. A total of 19 participants (14 OA, 5 HCs) aged 18–45 were recruited and grouped by allergy questionnaire score. Metabolites and lipids from tear samples were analysed using mass spectrometry. Data were analysed using TraceFinder and Metaboanalyst. Metabolomics analysis showed 12 differentially expressed (DE) metabolites between those with OA and the HCs during the peak allergy season, and 24 DE metabolites were found in the off-peak season. The expression of niacinamide was upregulated in OA sufferers vs. HCs across both seasons (*p* ≤ 0.05). A total of 6 DE lipids were DE between those with OA and the HCs during the peak season, and 24 were DE in the off-peak season. Dysregulated metabolites affected oxidative stress, inflammation, and homeostasis across seasons, suggesting a link between OA-associated itch and ocular surface damage via eye rubbing. Tear lipidome changes were minimal between but suggested tear film destabilisation and thinning. Such metabolipodome findings may pave new and exciting ways for effective diagnostics and therapeutics for OA sufferers in the future.

## 1. Introduction

Ocular Allergy (OA) is a localised form of allergy that typically peaks during spring-summer in Australia as the air becomes saturated with airborne allergens from pollen, fungal spores, animal dander, pollution, and dust. Increased outdoor activity and optimal weather for allergen spread mean that for the allergy sufferer, spring-summer can be detrimental to their ocular health and quality of life. Exposure to allergens on the ocular surface can trigger symptoms of OA, commonly including itchiness, irritation, redness and swelling of the eyelids, and conjunctiva [1]. While these symptoms may seem mild, emerging research has shown that allergy-induced itch and associated excessive eye rubbing may lead to physical changes to the ocular surface over time [2]. In fact, increased and sustained eye rubbing due to allergy has been shown to be a comorbidity to corneal ectasia, a progressive vision-degrading condition in which the cornea thins and sags [2]. This slow loss of vision can cause significant discomfort, blurry vision, itch, and irritation for keratoconus sufferers. In this case, the thinning and sagging may be exacerbated by allergy-associated eye rubbing, thus highlighting the need for new interventions for OA that prevent the primary symptom of itch on the ocular surface. To do so, it is crucial to investigate the biochemical changes occurring to the tear film and ocular surface as a result of OA to identify the baseline of metabolomic and lipidomic changes occurring during the peak allergy season and off-peak season.

To date, no studies investigating the human tear metabolome or lipidome of OA sufferers have been conducted, despite the significant proportion of Australia estimated to be suffering from seasonal allergies [3]. Instead, research has primarily investigated metabolomic biomarkers of allergic rhinitis, also known as hay fever, in serum and plasma [4,5,6]. While beneficial for determining how the metabolome may be impacted by allergic responses, serum and plasma metabolomic analysis is unsuitable for predicting how the ocular surface is impacted by OA. Instead, a direct snapshot of both metabolic and lipidomic activity on the ocular surface during peak allergy season could be captured by analysing the tears of OA sufferers, thereby providing a more focused insight into how the ocular surface and tear film are impacted by OA and how this may relate to symptom expression.

**Figure 1:**
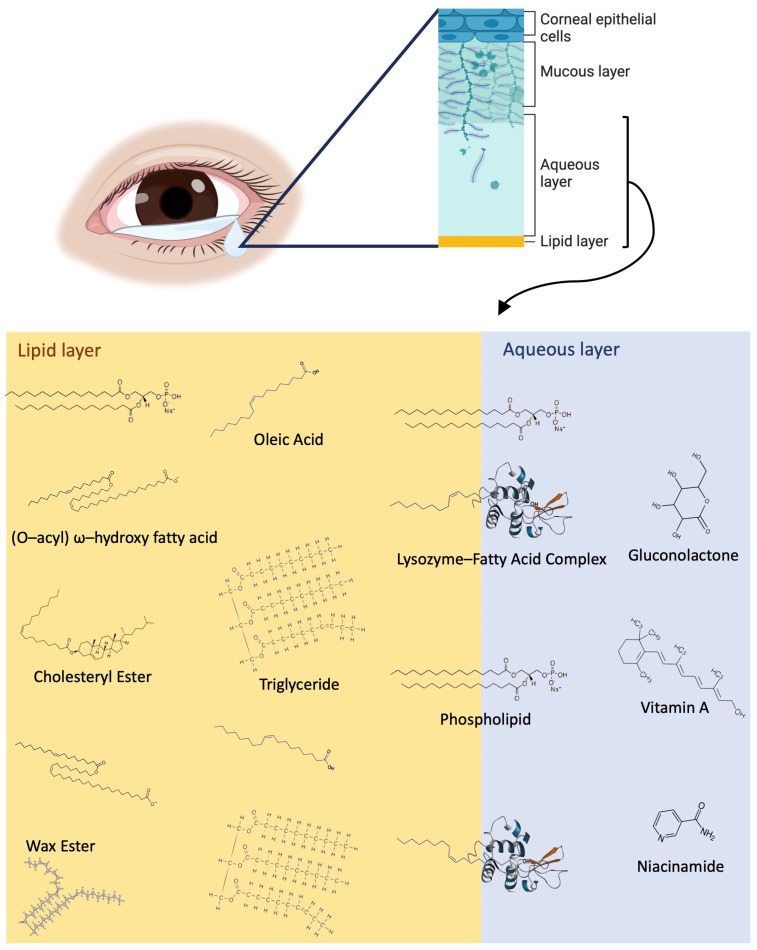
Overview of the tear film structure and a detailed view of the lipid and metabolite components of the lipid layer and aqueous layers, including fatty acids, WEs, OAHFAs, CEs, vitamin a, niacinamide, gluconolactone, and lysozymes. WEs, phospholipids, and CEs typically are closer to the surface of the tear film exposed to air, while fatty acids (represented by oleic acid) and triglycerides are distributed throughout the lipid layer. Lysozyme–fatty acid complexes cross the border between the aqueous and lipid layers, and the aqueous layer contains a variety of metabolites, including vitamin A, niacinamide, gluconolactone, and many more.

The tear film is comprised of three main layers: the innermost mucin layer, the intermediate aqueous layer, and the outermost lipid layer (Figure 1). The mucin layer and differential expression of mucins in OA sufferers tears have been reported in the past [7,8]. Metabolites, proteins, and electrolytes are abundant in the aqueous layer, and the lipid layer is rich with phospholipids, fatty acids, wax esters (WEs), (O-acyl) ω-hydroxy fatty acids (OAHFAs), triglycerides (TAGs), and cholesteryl esters (CEs), as shown in Figure 1.

Changes to the lipid and metabolite composition of the tear film in OA have not yet been investigated. However, studies of dry eye disease using human tear samples were able to detect increased oxidative stress and inflammation pathway activation through upregulated metabolites such as arginine and phenylalanine [9]. Despite the ability to detect changes to the metabolome in tears for ocular disorders, no studies of OA metabolomics using tears have been conducted to date. The most comparable studies are those using blood to analyse differentially expressed metabolites in allergic rhinitis and asthma sufferers.

However, a recent metabolomic study used serum and plasma samples to investigate changes to the metabolites during peak and off-peak allergy seasons among allergic rhinitis and asthma sufferers [10]. Another study by [11] used bronchiolar lavage fluid from pollen-sensitised mice to observe the effects of pollen exposure on oxidative stress and immune response in mice with stimulated allergic airway inflammation. These studies identified changes to the metabolome of season-allergy sensitised humans and mice relating largely to increased oxidative stress due to higher concentrations of pollen-derived Reactive Oxygen Species (ROS) and sustained periods of inflammation occurring over the allergy period [11]. No studies investigating the changes to the lipidome of OA sufferers, allergic rhinitis sufferers, or similar populations have been conducted to date.

The lack of studies in the literature relating to OA metabolomics and lipidomics highlight the highly novel nature of this study. The findings from this article provide a beneficial starting point for future studies aiming to determine how eye rubbing and inflammation may further change the ocular surface metabolome and lipidome of OA sufferers over time and thus could help design novel diagnostics and therapeutics for OA.

## 2. Results

Table 1 shows the number of participants, gender distribution, mean age, mean Quality of Life in Children with Vernal Keratoconjunctivitis (QUICK) questionnaire symptoms score, and mini Rhinoconjunctivitis Quality of Life Questionnaire (miniRQLQ) score across each of the four groups. No significant differences in gender ratios or age were found between the groups (*p* ≥ 0.05). Participants recruited during peak allergy season were categorised as OA and Healthy Controls (HCs) depending on their QUICK symptoms score and remained in the same grouping during the off-peak season to maintain a longitudinal study format. Participant dropouts between the peak allergy season and off-peak season were partially due to the significant amount of time between the seasons of 6 months, personal reasons such as illness or scheduling conflicts, and/or physiological factors such as the tear volume able to be collected.

The QUICK questionnaire findings showed that participants from the allergy group scored an average of 25.83 with a standard deviation (SD) of 19.30 during the peak allergy (PA) season and 24.40 (SD = 19.90) during the off-peak allergy (OPA) season, showing a slightly downward trend in symptom severity from the peak allergy season to off-peak season. The HCs’ maintained a mean score of 0 across both seasons (SD = 0).

The miniRQLQ scores were consistent with the grouping. Pearson’s correlation test between the miniRQLQ and QUICK symptoms scores indicated a moderately strong relationship (R^2^ = 0.63). The minimally significant value for each question was 0.7, meaning a cumulative score of below 9.8 across all 14 questions was negligible and does not indicate significant effects on QoL [12]. The mean miniRQLQ scores reported in HCs during the peak allergy season and off-peak allergy season were 9.76 (SD = 11.52) and 2.38 (SD = 2.38), respectively. The mean miniRQLQ score among OA sufferers was 33.84 (SD = 20.25) during the peak allergy season and 29.37 (SD = 18.35) during the off-peak season, highlighting a decreasing trend in QoL impacts during the off-peak season.

The OA sufferers, compared to HCs, showed significant effects of OA on desire to rub the eyes (peak allergy season *p* = 1.297 × 10^−5^; off-peak season *p* = 7.879 × 10^−5^) and itchiness (peak allergy season *p* = 6.810 × 10^−6^; off-peak season *p* = 2.00 × 10^−4^) and some effects on sleep quality (peak allergy season *p* = 0.009; off-peak season *p* = 0.024), as reported via the miniRQLQ.

### 2.1. Differential Expression of Metabolites and Lipids between Peak Allergy Season and Off-Peak Season

Twenty-seven metabolites were found to be significantly upregulated in the PA vs. peak allergy season healthy control (PHC) groups (*p* ≤ 0.05), and three were found to be significantly downregulated (*p* ≤ 0.05), as shown Supplementary data Table S1. An area under the curve (AUC) analysis was conducted on niacinamide as it was the only metabolite that was differentially expressed in both the peak allergy and off-peak seasons; therefore, it was theorised to be the most likely candidate to be a biomarker of OA.

In the OPA vs. off-peak season healthy control (OPHC) groups, 84 metabolites were significantly upregulated (*p* ≤ 0.05), and 3 were significantly downregulated (*p* ≤ 0.05), as shown in Table S1. No lipids were found to be downregulated between the OPA and OPHC groups. Of the upregulated lipids, Type II diesters were the most abundant and significantly differentially expressed class.

### 2.2. Peak Data ROC Curves

**Figure 2:**
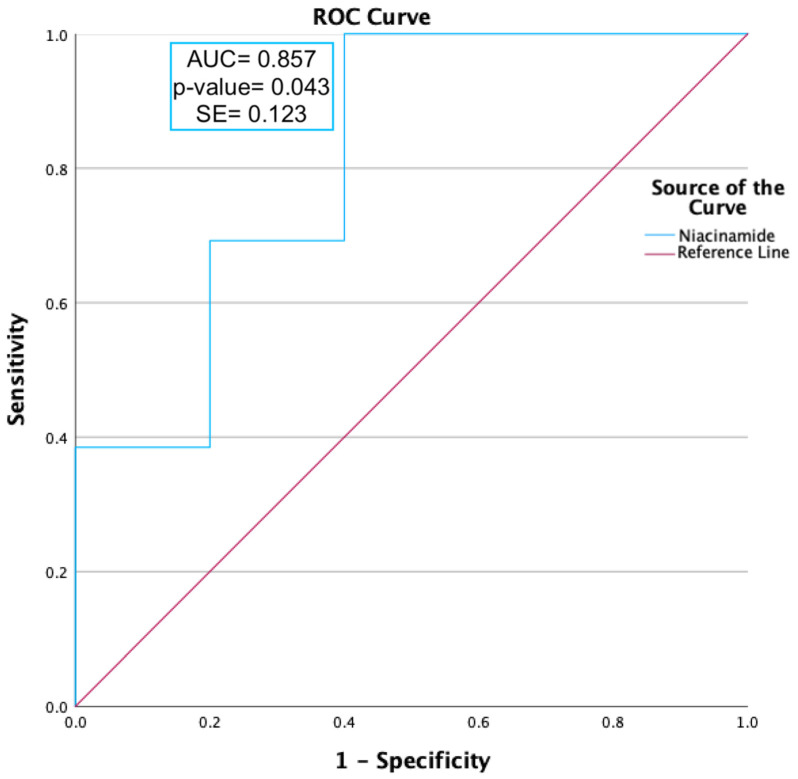
ROC curve analysis of niacinamide expression during peak allergy season between ocular allergy sufferers (PA) and healthy controls (PHCs). Niacinamide had an area under the curve (AUC) value of 0.86 and a *p*-value of 0.043, indicating a moderate ability to predict OA status.

ROC curve analysis was carried out on niacinamide, as shown in Figure 2. Niacinamide (*p*-value = 0.012) was found to have a moderately predictive capability, with an AUC value ≥ 0.85.

### 2.3. Functional Analysis of Upregulated Metabolites

**Figure 3:**
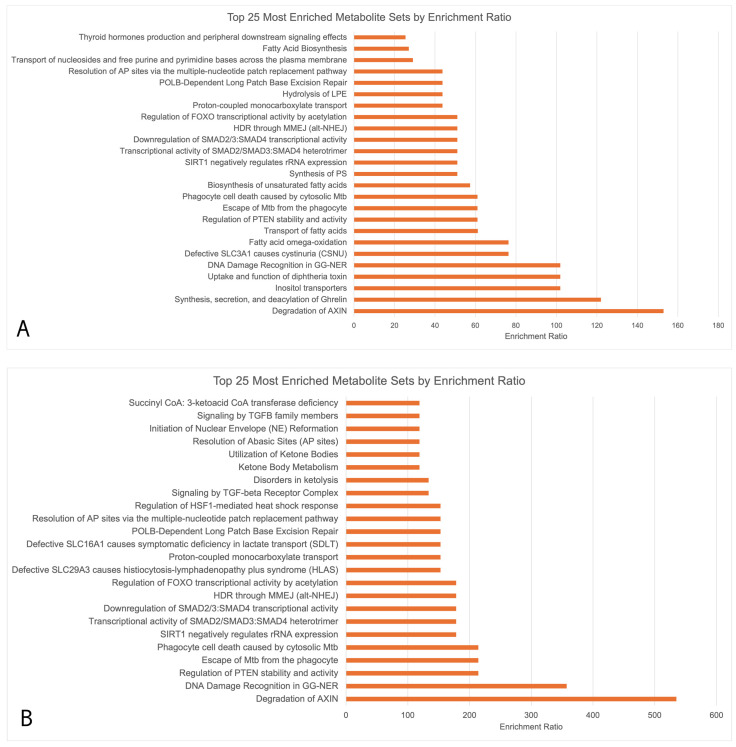
MetaboAnalyst enrichment analysis conducted using RaMP consisting of HMDB, KEGG, Reactome, and WikiPathways simultaneously. Only upregulated metabolites underwent enrichment analysis due to the low numbers of downregulated metabolites (<5 during both peak allergy season and off-peak season). (**A**) Upregulated metabolites in PA vs. PHCs; (**B**) upregulated metabolites in OPA vs. OPHCs.

Enrichment analysis (Figure 3) showed that the upregulated metabolites in Figure 3A,B had 13 key overlapping pathways in OA sufferers vs. HCs across both seasons, including the degradation of the axin protein, DNA damage recognition in Genome Nucleotide Excision Repair (GG-NER), the regulation of Phosphatase and Tensin Homolog (PTEN) stability and activity, transcriptional activity of signal-transducing smad protein, the downregulation of smad, and the regulation of Forkhead Box (FOXO) transcriptional activity by acetylation. Downregulated biopathways during both the peak allergy and off-peak seasons did not undergo functional enrichment analysis due to the small number of downregulated metabolites (>5 in both seasons). The upregulated pathways were largely related to the metabolism of amino acids.

### 2.4. Differentially Expressed Metabolites by Allergy Status

#### 2.4.1. PA/OPA

In the allergy groups (PA vs. OPA), across both seasons, 11 metabolites were found to be differentially expressed, as shown in Supplementary data Table S2. Among these, a diglyceride (DAG) lipid (*p*-value = 0.005; Log_2_FC = +3.514), glycerol (*p*-value = 0.015; Log_2_FC = +3.613), and fructose-1-phosphate (*p*-value = 0.022; Log_2_FC = +2.601) were the most significantly differentially expressed.

#### 2.4.2. PHC/OPHC

In the control groups (PHC vs. OPHC), across the seasons, 38 total metabolites were differentially expressed. Table S2 shows the significance, relative abundance, and direction of differential expression between the groups for each significantly differentially expressed lipid and metabolite.

## 3. Discussion

### 3.1. Questionnaire Data

The questionnaire-assessed QoL changes identified in this study show the significant negative impact of OA on QoL for allergy sufferers. OA sufferers reported negative QoL effects in both the peak allergy season and off-peak season, as evidenced by a consistently high mean miniRQLQ score of 33.84 (SD = 20.25) during the peak allergy season and 29.37 (SD = 18.35) during the off-peak season. Furthermore, the questionnaire data in this study show that OA significantly reduces sleep quality all year round (peak allergy season *p* = 0.009; off-peak season *p* = 0.024) and leads to a consistently increased desire to rub the eyes (peak allergy season *p* = 1.297 × 10^−5^; off-peak season *p* = 7.879 × 10^−5^) irrespective of the season. QoL changes associated with OA were shown to be experienced all year long, indicating that there may be equally long-lasting metabolomic and lipidomic effects of OA occurring on the ocular surface. Investigating longitudinal metabolomic and lipidomic changes occurring on the ocular surface due to OA is the novel concept driving this study.

In this study, we characterised the human tear lipidome and metabolome of OA sufferers vs. HCs across the peak allergy season and off-peak season for the first time. In doing so, we found significant differentially expressed metabolites relating to oxidative stress, DNA damage, and inflammation on the ocular surface. In addition, lipidomics analysis showed significant effects of OA and seasonal variation on the stability and structure of the tear film. Together, these findings indicate that seasonal variations in the metabolome and lipidome of the tears are able to detect key changes to the ocular surface that greatly affect symptom expression and the return to homeostasis.

### 3.2. Comparisons between OA and HCs in Peak Allergy and Off-Peak Seasons

In the peak allergy season, upregulated metabolites were indicative of ocular surface biopathways attributed to inflammation, wound healing, and oxidative stress regulation resulting from allergy pathway activation. Ethanolamine (Log_2_FC = +1.974), gluconolactone (Log_2_FC = +4.384), an OAHFA species (54:0) (Log_2_FC = +3.430), a CE species (27:1) (Log_2_FC = +4.365), and threonic acid (Log_2_FC = +2.129) were the five most significantly upregulated metabolites (Table S1). Enrichment analysis (Figure 3A) suggested that the general functional associations of these upregulated metabolites were stem cell differentiation, axin protein degradation, GG-NER, the regulation of PTEN stability/activity, and increased oxidative stress.

Oxidative stress is a cellular phenomenon in which redox balance is disturbed by excess ROS in the cell, resulting in cell and tissue damage [10]. Oxidative stress occurs on the ocular surface as a result of inflammation and pollen exposure and has been implicated in a variety of atopic disorders, including asthma and allergic rhinitis, in the past [10]. Oxidative stress responses occur due to exposure to NAPDH oxidase enzymes from pollen granules entering ocular surface cells and triggering the production of ROS [11]. ROS can then dysregulate cell signalling pathways and lead to apoptosis, immune system hyperactivity, and DNA damage [11]. In our study, GG-NER DNA damage regulation pathways were shown, for the first time, to be activated on the ocular surface of OA sufferers compared to HCs, as indicated by an increase in related metabolites in both the peak allergy and off-peak seasons (Figure 3A,B). PTEN and GG-NER have been associated with oxidative damage in epithelial cells, and in this context, they may indicate the DNA- and tissue-level healing and repair processes occurring on the ocular surface (commonly referred to as wound healing) [13].

The enrichment analysis in this study showed increased PTEN stability and activity regulation, likely referring to the inhibition of PTEN on the ocular surface and the subsequent activation of the phosphoinositide 3-kinase (PI3K) and serine/threonine protein kinase B (Akt) signalling pathway (PI3K/Akt pathway) [14]. The presence of upregulated metabolites from this biopathway in OA sufferers versus HCs across both seasons therefore infers an increase in wound healing, potentially increasing the migration of epithelial cells to sites of OA-induced itch and eye rubbing to induce tissue-level repairs to the ocular surface [14]. Epithelial cell transport and inflammation in allergy-affected immune cells both significantly increase the energy production requirements of the cell, therefore indicating that there must be metabolic changes occurring on the ocular surface that have a net increase in energy production.

The activation of the PI3K/Akt pathway has also been shown to trigger the Warburg effect in immune cells, an effect responsible for modifying energy production in immune cells. This effect dysregulates glycolysis and energy production pathways (via the alteration of the Krebs cycle), which ultimately increases ROS production and, thus, oxidative stress in immune cells while also reducing effective energy production [15]. In T-helper cells, this energy loss is compensated for by the overproduction of glutamate, an alternate input source for the Krebs cycle that leads to a net boost in energy production. In antigen-presenting cells, energy production is decreased due to alterations to the Krebs cycle that reduce energy output while increasing ROS formation and, thus, oxidative stress. Oxidative stress can be managed by the expression of metabolic mediators such as the Forkhead Box (FOXO) transcription regulator. FOXO expression was found to be positively associated with increased ROS in endothelial cell culture models of oxidative stress [16]. In this study, FOXO was found to increase the expression of antioxidants, balancing ROS and thus reducing the effects of oxidative stress [15]. Thus, it was indicated that, in OA sufferers, compared to HC across both seasons, FOXO regulation was likely to increase in order to alleviate oxidative stress on the ocular surface while stimulating angiogenesis in response to allergen exposure. Angiogenesis is the formation of new capillaries and has been heavily implicated in OA in the past as being the underlying cause of two of the key symptoms of OA, redness and swelling [17]. This process is theorised to be upregulated during and after allergic responses in order to facilitate the passage of cell signalling molecules, immune cells, and other factors on the ocular surface [17]. Angiogenesis increases the efficacy of wound healing factor transport to sites of inflammation and injury on the ocular surface. It is therefore theorised that wound healing biopathways have a crucial impact on the ocular surface of OA sufferers to facilitate the return to homeostasis.

Wound healing is the process by which cells regenerate and heal following cellular stress, likely also relating to the stem cell differentiation biopathway highlighted in Figure 3A. In human corneal epithelial cells, the PI3K/Akt pathway leads to enhanced wound healing by encouraging the targeted migration of epithelial cells to assist with the release of wound healing mediators at affected sites [14]. These repair and wound healing processes typically involve the generation of new cells to replace damaged and broken-down cells, as evidenced by the identification of stem cell differentiation as another key affected biopathway.

Stem cell differentiation and axin protein degradation (Figure 3A,B) may also be indicative of increased cell proliferation as a result of Wnt signalling pathway inhibition on the ocular surface [18]. Axin is a key component of the Wnt signalling pathway, in which cell proliferation, apoptosis, and homeostasis are blocked by the axin-induced formation of the β-catenin destructive complex [18]. Axin degradation was significant in both peak allergy and off-peak season allergy sufferers compared to HCs, thus highlighting increased cell turnover via Wnt signalling pathway inhibition, possibly to combat inflammation and oxidative stress on the ocular surface [18].

These anti-inflammatory mediators are important, as they play a crucial role in the body’s immune response by compensating for increased inflammation resulting from allergy pathway activation. Inflammation was shown to be affected by errant metabolic biopathway activation, as evidenced by the increased negative regulation of smad proteins identified in the enrichment analysis of both peak allergy and off-peak season OA sufferers compared to the HCs (Figure 3A,B). On the ocular surface, the negative regulation of signal-transducting smad proteins would likely lead to a decreased expression of Tumour Growth Factor β (TGF-β) cytokine. TGF-β expression has been highlighted in previous studies as a key anti-inflammatory cytokine [19], therefore indicating a possible net increase in inflammation regulation on the ocular surface of OA sufferers irrespective of the season.

Intriguingly, niacinamide was the only metabolite shown to be consistently differentially expressed in both peak (Log_2_FC = 2.148) and off-peak (Log_2_FC = 1.68) OA sufferers compared to the HCs. Niacinamide (AUC = 0.877; *p* = 0.016) was capable of predicting OA status (asymptomatic significance ≤ 0.05), as shown in Figure 2, and thus must be significantly linked to the biopathways of OA. The effects of the upregulation of niacinamide have not been reported in any ocular studies; however, it has been shown in studies of skin to have a net anti-inflammatory effect through the inhibition of the Nuclear Factor-kappa-light-chain-enhancer of activated B cells (NF-κB) biopathway [20]. NF-κB is a transcriptional factor that affects the expression of pro-inflammatory cytokines and chemokines in cells [20]. Additionally, niacinamide has been theorised to be involved in cell rebuilding by influencing the expression of keratins and differentiation of structural keratinocytes, likely occurring here to compensate for allergy-induced itch and eye rubbing [21].

It is theorised that in this context, niacinamide metabolite secretion is upregulated due to anti-inflammatory cellular pathway activation occurring on the ocular surface of OA sufferers vs. HCs, irrespective of the season. Similarly, nicotinamide adenine dinucleotide (NAD+) biosynthesis (Figure 3A,B) and its associated metabolites form a network responsible for the regulation of gene expression, energy metabolism, and DNA damage repair in response to increased oxidative stress and inflammation in the local environment [22]. This biosynthetic pathway was uniquely increased in off-peak OA sufferers compared to HCs. An increased expression of theanine metabolite (Log_2_FC = 1.50) may also contribute to inflammation in the off-peak season, as it has been shown to have an anti-allergy effect by preventing the expression of allergic cytokines such as TNF-α, IL-1β, IL-6, and IL-8 via the inhibition of the NF-κB biopathway, occurring in allergy-mediating mast cells [23]. It was therefore theorised that lipid expression differences may impact inflammation in addition to metabolites; however, this was not the case in this study.

The tear film is crucial for maintaining a lubricated ocular surface to prevent dry eye disease, irritation, and infection [24]. Decreased lipid concentrations in the tears may instead be related to increased lipid and amino acid metabolism caused by the downregulation of homogentisic acid (Log_2_FC = −5.620), hexanoylglycine (Log_2_FC = −3.262), and ornithine (Log_2_FC = −2.110). A study from 2019 investigated serum metabolite differences in hay fever sufferers during peak allergy and off-peak seasons, finding a decreased expression of ornithine and histidine [25]. Both were said to likely be involved in amino acid metabolism [25]. A study of non-seasonally linked allergic rhinitis caused by house dust mites found that gluconolactone was downregulated between mild and severe subgroups of allergic rhinitis, suggesting that gluconolactone’s involvement in amino acid metabolism is downregulated in severe allergy participants [5]. The findings from our study of OA sufferers vs. HCs are consistent with those of [25], with ornithine reportedly being downregulated in the peak allergy season. Gluconolactone was upregulated between the OA and HC groups during the peak allergy season (Log_2_FC = +4.38) in this study, which contradicts the findings reported by [5] possibly due to different metabolic mechanisms being employed in non-seasonally linked allergic rhinitis compared to seasonal OA. Another 2019 study by [4], which looked at serum metabolomics of allergic rhinitis patients, found that arachidonic acid metabolism was affected by allergic rhinitis [4]. Arachidonic acid metabolism generally refers to the biopathway in which eicosanoid lipid mediators influence inflammation in the body [4]. Evidence of this was not reported by our allergy vs. HC findings and was not conclusively reported in [4], with reported metabolites and lipids indicating pro- and anti- inflammatory pathways being expressed simultaneously in the serum of allergy sufferers compared to HCs [4]. Differentially expressed lipids are thereby theorised to play differing roles in tear film stability depending on species and the direction of differential expression.

In the PA group versus the PHCs of our study, no lipids were significantly downregulated; however, five were found to be upregulated. Overall, this displays a general increase in lipid expression on the ocular surface between peak season OA sufferers and HCs. This increase in lipid expression may occur to compensate for tear evaporation due to allergy-associated dry eye.

Type I and II diesters (DE-I and DE-II) were the largest dysregulated class of lipids found between the OPA and OPHC groups, followed by OAHFAs, CEs, WEs, PCs, and TAGs. These lipids were all upregulated among the OPA vs. OPHC groups, indicating increases in tear film stability, structure, and antimicrobial activity, characteristic of tear film regeneration and the return to homeostasis [26,27]. Linking these findings to the metabolomics, we hypothesise that there is a continual effect of allergy on the tear film from the peak allergy season into the off-peak season. This finding is illustrated best by the upregulation of stabilising lipids such as OAHFAs and WEs to compensate for persistent oxidative stress and inflammation on the ocular surface, even in the off-peak season.

### 3.3. Comparisons by Allergy Status across Seasons

Very few changes occurred in OA sufferers between the peak and off-peak seasons, potentially due to consistent inflammation and oxidative stress occurring across seasons, as indicated by lipidomics data and the differentially expressed metabolites between the OA and HC groups in the off-peak season.

Among the HCs, 14 OAHFA species were upregulated during the peak allergy season compared to the off-peak season, as well as 4 WE species. OAHFAs and WEs are structurally similar lipids that may work in conjunction to maintain the tear film and tear film lipid layer by acting as surfactants stabilising the tear film across the ocular surface [27]. This is indicated by their presence in the uppermost portion of the lipid layer of the tear film, shown in Figure 1. Conversely, the downregulation of PCs, a PE, and an SM indicate an overall decrease in lipid layer homeostasis, surface tension, and a susceptibility to oxidative damage on the ocular surface [26,27,28]. The HCs showed significant differential expressions of OAHFAs between the peak and off-peak season, which may be present to enact an anti-inflammatory effect in conjunction with the downregulation of eicosapentaenoic acid during the peak allergy season (Log_2_FC = −2.48) [4,27].

The key limitation of this study is the low sample size representing each group, particularly during the off-peak season. This study was an explorative pilot longitudinal investigation of metabolomic and lipidomic changes to the tear film in OA sufferers vs. HCs. Due to the longitudinal study design, participants recruited during the peak allergy season were required to return in the off-peak season. However, due to the 6-month gap between sessions, participant dropouts due to scheduling difficulties led to a reduced sample size. Additionally, the tear volume able to be collected in the peak allergy season and off-peak season was not consistent, with some participants no longer able to provide a sample due to ocular surface dryness. This dry eye was not necessarily caused by OA during the winter, as the reduced number of tear samples (*p* > 0.05) may have been due to external factors such as screen time or the time of day. As a result, sample numbers were decreased during the off-peak season. The significance of the data was still high. Enrichment analysis using RaMP has been used in previous metabolomics studies [29,30,31] and in this study, it was used to provide an overview of the most likely impacted metabolic pathways; however, additional data filtering by the selection of statistically significant connections (*p* < 0.05) must be implemented to infer meaningful connections to findings. Ultimately, findings should be validated in a large-scale cohort study prior to diagnostic or therapeutic development based on results from this study.

## 4. Methods and Materials

### 4.1. Study Population

This study was conducted in accordance with research ethics guidelines, as advised by the Deakin University Human Research Ethics Committee (DUHREC study #2021-189). The study population consisted of 19 adults aged 18–45 from Victoria, Australia. Written informed consent was obtained from all participants prior to study commencement. Overall, 14 OA sufferers and 5 HCs were included in the study.

### 4.2. Clinical Exam Sequence

Participants were asked to attend two sessions, one during the peak allergy season (spring-summer) and another during the winter during the off-peak allergy season, in which they were provided with questionnaires assessing participant demographics, ocular health history, and symptom severity assessments.

Best-corrected visual acuity and ocular health were assessed using an electronic LogMAR chart and slit lamp with the Efron grading scale [32] before and after sample collection to ensure no changes to ocular health resulted from the collection process. Fluorescein dye and slit lamp assessment of the ocular surface was used to detect any potential irritation caused by sample collection at the end of each session.

### 4.3. Questionnaires

Participant eligibility was determined by adherence to inclusion and exclusion criteria of age (18–45 years old), ocular health history (no recent reported infections, injury or surgery), and other health factors (pregnancy/breastfeeding). Other forms of atopy, including asthma or atopic dermatitis, were noted but not used as exclusion criteria.

The QUICK questionnaire symptom severity scores were converted from 0–6-point responses to a singular 0–100 score using the following formula:

(Total score/(highest possible score − lowest possible score)) × 100

Symptom scores of zero were used to identify and classify control participants, as these scores indicated the absence of OA symptoms. Scores greater than 1 classified participants as OA sufferers [33].

The miniRQLQ was also administered in order to gauge quality of life effects of OA in greater detail. This questionnaire consisted of 14 questions graded from 0 to 6.

Correlation, mean, and standard deviation statistical analyses on questionnaire data were carried out using IBM SPSS software version 29.0.0.0 (IBM, Armonk, NY, USA).

### 4.4. Tear Sample Collection

Tear samples were collected using the glass microcapillary flow technique [33]. Regarding tear samples, 5–45 μL of tears were collected and pooled from the outer corner of each eye. The samples were transferred to a −80 °C freezer for storage.

### 4.5. MTBE Metabolite and Lipid Extraction

To extract metabolites and lipids from tears, a two-phase liquid/liquid MTBE extraction protocol was used [34]. To do this, 20 μL of each tear sample was aliquoted into a 1.5 mL Eppendorf tube, to which a solution of 200 μL of ice-cold methyl tert-butyl ether (MTBE) containing 1:100 SPLASH lipidomics internal standard mix (Avanti, Alabaster, AL, USA) was added. Following this, 60 μL of methanol was added, containing the metabolite internal standards 20 µM ^13^C_3_^15^N-alanine/D_4_Cit. Samples were then extracted on an orbital shaker at 950 rpm for 1 h at room temperature. The samples were then centrifuged at maximum speed (13,500 rpm) for 5 min at room temperature. After centrifugation, the tear samples were separated into two distinct layers, an upper organic/lipid layer and a low aqueous/metabolite containing layer with a protein pellet at the bottom.

### 4.6. Gas Chromatography–Mass Spectrometry (GC-MS) Polar Metabolite Derivatisation

A 50 μL aliquot of the lower aqueous layer was transferred to a fresh Eppendorf tube. The sample was then dried on a SpeedVac for 30 min at room temperature. Once dried, 20 μL of 30 mg/mL MeOX in pyridine was added to the tube, which was then incubated at 55 °C for 30 min at 950 rpm on a ThermoMixer (Eppendorf, Hamburg, Germany). Finally, 25 μL of TMS was added, and the mixture was incubated for 30 min at 37 °C.

### 4.7. GC-MS Metabolite Analysis

Samples were analysed using gas chromatography–triple quadrupole mass spectrometry (GC-MS, Shimadzu TQ8040, Kyoto, Japan). The Shimadzu metabolomics MRM library was used; MRMs were scheduled by running an alkane mix (Restek qualitative retention time index standard). A 30 m × 0.25 mm id, 1 µm film DB-5 (Agilent, Santa Clara, CA, USA) column was used for separation using He as the carrier gas at 1 mL/min constant flow. The temperature program commenced at 100 °C, a temperature which was held for 4 min. The oven temperature then increased at 10 °C/min until 320 °C. The column was held at 320 °C for 11 min. The MS transfer was 280 °C, and the ion source temperature was 200 °C. A 1 µL aliquot of each sample was injected in splitless mode with an inlet liner temperature of 250 °C. Peak areas were determined using Lab Solutions Insite software version 5.91 (Shimadzu).

### 4.8. LC-MS Lipidomics

A 150 µL aliquot of the MTBE layer for each sample was transferred to a 200 µL vial insert. These were then placed in Eppendorf tubes and dried in a speed vacuum concentrator. After drying, the inserts were transferred to LC vials, and 50 µL of isopropyl alcohol was added. The vials were sonicated for 10 min to aid the dissolution of lipids.

Lipids were analysed in duplicate by LC-MS using a Vanquish flex UPLC system coupled to an Orbitrap Exploris-240 high-resolution mass spectrometer. A 2.1 × 50 cm, 1.9 µm C18 LC column (Hypersil gold ThermoScientific, Waltham, MA, USA) was used with a binary mobile phase at a flow rate of 0.4 mL/min. The column temperature was 50 °C. Mobile phase A was water with 10 mM ammonium formate; mobile phase B was water/acetonitrile/isopropanol (5:20:75) with 10 mM ammonium formate. The mobile phase composition commenced at 50%B; this was ramped to 100%B over 10 min, held at 100% B for 3 min, and then re-equilibrated at 50%B for 2 min.

The MS was operated in positive-ion data-dependant MS/MS mode. Full scan was from 200 to 2000 *m*/*z* at 120,000 resolution with easy-IC internal mass calibration; the MS/MS mode resolution was 15,000 (easy IC off), with normalised collision energies % of 20, 40, 60 being used.

The peak areas of target species were determined using Tracefinder software version 5.1 (Thermo, Waltham, MA, USA). The retention time, accurate mass, and MS/MS spectra were used to confirm the identification of lipid species.

### 4.9. Statistical Analysis

Peak area data were normalised by input volume as the volume of tear samples used for MTBE extraction varied between 10 and 20 µL total. Once normalised, the data were then standardised by dividing by the expression of the internal standards. In the metabolites, the internal standard used was alanine, and for lipids, the standard was the 15:0-18:1(d7)-15:0 TAG.

Standardised data for lipids and metabolites were separately analysed for differential expression analyses using MetaboAnalyst 5.0 (https://www.metaboanalyst.ca/home.xhtml (accessed on 15 January 2024)). The one-factor statistical analysis mode was used. Data were log_10_ transformed and mean-centred to normalise their distribution. Orthogonal least partial squares discriminant analysis (OPLS-DA) and two-way Welch’s *t*-tests assuming unequal variances were conducted on the normalised data to determine differentially expressed lipids and metabolites between the OA and HC groups in both the peak allergy and off-peak seasons. The Variable Importance in Projection (VIP) value was obtained through OPLS-DA analysis. Differential expression was reported as log_2_ fold change (Log_2_FC) values and *p*-values. Resultant differential expression data were filtered for statistical significance (*p* ≤ 0.05), VIP score > 1 and a Log_2_FC of ±1.5.

Additionally, differentially expressed metabolites and lipids between the OA and HC groups during the peak allergy season were further assessed for allergy status predictive ability using IBM SPSS software version 29.0.0.0 (IBM). Area under the curve (AUC) analysis was conducted on differentially expressed metabolites of interest. AUC values of greater than 0.85 were deemed significant, and receiver operating characteristic (ROC) curve analysis was conducted to assess the viability further. Metabolites with a *p*-value of *p* ≤ 0.05 identified by ROC curve analysis were considered as able to predict OA status and were thus highlighted as likely candidates for OA biomarkers.

### 4.10. Enrichment Analysis

Enrichment analysis was also conducted on MetaboAnalyst using KEGG IDs for differentially expressed metabolites. Upregulated and downregulated metabolites were analysed separately for each season. RaMP enrichment analysis [29,30,31,32,33,34] was used to map differentially expressed metabolites to HMDB, KEGG, Reactome, and WikiPathways simultaneously. A *p*-value ≤ 0.05 was considered a significant association to biological pathways.

## 5. Conclusions

This study identified the errant expression of niacinamide occurring in allergy sufferers irrespective of the season, which is theorised to be related to rebuilding structural networks in the cells by promoting the expression of keratins, as shown in the skin. Unfortunately, metabolomics findings were unable to be fully explored due to insufficient research on the allergy-affected metabolic pathways of OA and the specific roles of differentially expressed metabolites on the ocular surface. In this study, niacinamide was shown to have high OA prediction capacity, but its role has not been defined in ocular surface immunometabolomics research thus far. In order to develop and interpret these findings in a diagnostic capacity, additional studies looking at the interactions between niacinamide and other tear constituents, such as proteins, cytokines, and lipids, will be crucial. Metabolomics analysis was, however, able to highlight the significant impact of oxidative stress and inflammation resulting from ROS generation and allergen exposure on the ocular surface.

This study also aimed to investigate the human tear lipidome in ocular allergy sufferers for the first time, ultimately finding that tear film stability is dysregulated between peak season allergy sufferers and HCs. However, the more compelling findings showed that tear film stability is even more dysregulated by seasonal influence, as determined by the differential expression of lipids among the HCs.

This may highlight the likely protective role of the tear lipidome on the ocular surface, preventing significant differences in the expression of lipids among OA sufferers. The differences in lipid expression among the HCs in the peak allergy season compared to the off-peak season are stark and paint the image of a lipidome capable of adjusting to the needs of the ocular surface depending on external weather conditions. In Australia, the winter months tend not to be as cold as in other parts of the world but are more windy, potentially influencing the high incidence of dry eye and thus causing the overexpression of tear film-stabilising lipids such as OAHFAs and WEs to compensate [35].

The lipidomes and metabolomes of OA sufferers compared to HCs, assessed across multiple seasons, have never been studied before. This research is highly novel and highlights several potential links to differentially expressed proteins on the ocular surface and the biopathways they may influence. As a result, this study has provided invaluable insight into what takes place on the ocular surface during peak allergy season and how this may change over time.

## Figures and Tables

**Table 1:** Participant demographics. No significant differences were observed in age or mean protein concentration between the groups (*p* ≤ 0.05). The QUICK symptom score showed a downward trend in allergy symptom scoring between the peak allergy season and off-peak allergy season in ocular allergy sufferers. PA: peak season allergy sufferers; PHCs: peak allergy season healthy controls; OPA: off-peak season allergy sufferers; OPHCs: off-peak season healthy controls.

Characteristic	Peak Allergy Season		Off-Peak Season	
	Allergy Group (PA)	Control Group (PHCs)	Allergy Group (OPA)	Control Group (OPHCs)
Number of participants	14	5	7	3
Number of females/males	10:4	2:3	5:2	2:1
Age (mean ± standard deviation)	32.71 ± 7.65	28.20 ± 2.17	31.71 ± 8.65	27.67 ± 2.08
QUICK symptoms score (mean ± standard deviation)	25.83 ± 19.30	0 ± 0	24.40 ± 19.90	0 ± 0
miniRQLQ score (mean ± standard deviation)	33.84 ± 20.25	9.76 ± 11.52	29.37 ± 18.35	2.38 ± 2.38

## Data Availability

The datasets presented in this article are not readily available due to ethical concerns regarding participant confidentiality. Requests to access the datasets should be directed to cenk.suphioglu@deakin.edu.au.

## Supplementary Materials

The supporting information can be downloaded at: https://www.mdpi.com/article/10.3390/ijms25074019/s1.

## References

[1] Leonardi A., Castegnaro A., Valerio A.L.G., Lazzarini D. (2015). Epidemiology of allergic conjunctivitis. Curr. Opin. Allergy Clin. Immunol..

[2] McMonnies C.W. (2009). Mechanisms of Rubbing-Related Corneal Trauma in Keratoconus. Cornea.

[3] Beggs P.J., Katelaris C.H., Medek D., Johnston F.H., Burton P.K., Campbell B., Jaggard A.K., Vicendese D., Bowman D.M., Godwin I. (2015). Differences in grass pollen allergen exposure across Australia. Aust. N. Z. J. Public Health.

[4] Ma G., Wang T., Wang J., Ma Z., Pu S. (2019). Serum metabolomics study of patients with allergic rhinitis. Biomed. Chromatogr..

[5] Xie S., Zhang H., Xie Z., Liu Y., Gao K., Zhang J., Xie S., Wang F., Fan R., Jiang W. (2021). Identification of Novel Biomarkers for Evaluating Disease Severity in House-Dust-Mite-Induced Allergic Rhinitis by Serum Metabolomics. Dis. Markers.

[6] Zheng P., Yan G., Zhang Y., Huang H., Luo W., Xue M., Li N., Wu J.-L., Sun B. (2021). Metabolomics Reveals Process of Allergic Rhinitis Patients with Single- and Double-Species Mite Subcutaneous Immunotherapy. Metabolites.

[7] Dogru M., Okada N., Asano-Kato N., Igarashi A., Fukagawa K., Shimazaki J., Tsubota K., Fujishima H. (2006). Alterations of the ocular surface epithelial mucins 1, 2, 4 and the tear functions in patients with atopic keratoconjunctivitis. Clin. Exp. Allergy.

[8] Dogru M., Matsumoto Y., Okada N., Igarashi A., Fukagawa K., Shimazaki J., Tsubota K., Fujishima H. (2008). Alterations of the ocular surface epithelial MUC16 and goblet cell MUC5AC in patients with atopic keratoconjunctivitis. Allergy.

[9] Galbis-Estrada C., Martinez-Castillo S., Morales J.M., Vivar-Llopis B., Monleón D., Díaz-Llopis M., Pinazo-Durán M.D. (2014). Differential Effects of Dry Eye Disorders on Metabolomic Profile by1H Nuclear Magnetic Resonance Spectroscopy. BioMed Res. Int..

[10] Sagdic A., Sener O., Bulucu F., Karadurmus N., Özel H., Yamanel L., Tasci C., Naharci I., Ocal R., Aydin A. (2011). Oxidative stress status and plasma trace elements in patients with asthma or allergic rhinitis. Allergol. Immunopathol..

[11] Boldogh I., Bacsi A., Choudhury B.K., Dharajiya N., Alam R., Hazra T.K., Mitra S., Goldblum R.M., Sur S. (2005). ROS generated by pollen NADPH oxidase provide a signal that augments antigen-induced allergic airway inflammation. J. Clin. Investig..

[12] Juniper E., Thompson A.K., Ferrie P.J., Roberts J.N. (2000). Development and validation of the mini Rhinoconjunctivitis Quality of Life Questionnaire. Clin. Exp. Allergy J. Br. Soc. Allergy Clin. Immunol..

[13] Ming M., Feng L., Shea C.R., Soltani K., Zhao B., Han W., Smart R.C., Trempus C.S., He Y.-Y. (2011). PTEN Positively Regulates UVB-Induced DNA Damage Repair. Cancer Res.

[14] Cao L., Graue-Hernandez E.O., Tran V., Reid B., Pu J., Mannis M.J., Zhao M. (2011). Downregulation of PTEN at Corneal Wound Sites Accelerates Wound Healing through Increased Cell Migration. Investig. Opthalmol. Vis. Sci..

[15] Goretzki A., Zimmermann J., Lin Y.-J., Schülke S. (2022). Immune Metabolism–An Opportunity to Better Understand Allergic Pathology and Improve Treatment of Allergic Diseases?. Front. Allergy.

[16] Wan S., Pan Y., Yang W., Rao Z., Yang Y. (2020). Inhibition of EZH2 alleviates angiogenesis in a model of corneal neovascularization by blocking FoxO3a-mediated oxidative stress. FASEB J..

[17] Lee H.-S., Hos D., Blanco T., Bock F., Reyes N.J., Mathew R., Cursiefen C., Dana R., Saban D.R. (2015). Involvement of Corneal Lymphangiogenesis in a Mouse Model of Allergic Eye Disease. Investig. Opthalmol. Vis. Sci..

[18] Zhang Y., Yeh L.-K., Zhang S., Call M., Yuan Y., Yasunaga M., Kao W.W.-Y., Liu C.-Y. (2015). Wnt/β-catenin signaling modulates corneal epithelium stratification via inhibition of Bmp4 during mouse development. Development.

[19] Boehm N., Riechardt A.I., Wiegand M., Pfeiffer N., Grus F.H. (2011). Proinflammatory Cytokine Profiling of Tears from Dry Eye Patients by Means of Antibody Microarrays. Investig. Opthalmol. Vis. Sci..

[20] Grange P.A., Raingeaud J., Calvez V., Dupin N. (2009). Nicotinamide inhibits Propionibacterium acnes-induced IL-8 production in keratinocytes through the NF-κB and MAPK pathways. J. Dermatol. Sci..

[21] Tanno O., Ota Y., Kitamura N., Inoue S. (1997). Effects of niacinamide on ceramide biosynthesis and differentiation of cultured human keratinocytes. J. Investig. Dermatol..

[22] Kang H., Park Y.-K., Lee J.-Y. (2021). Nicotinamide riboside, an NAD+ precursor, attenuates inflammation and oxidative stress by activating sirtuin 1 in alcohol-stimulated macrophages. Mod. Pathol..

[23] Kim N.H., Jeong H.J., Kim H.M. (2011). Theanine is a candidate amino acid for pharmacological stabilization of mast cells. Amino Acids.

[24] Shimmura S., Ueno R., Matsumoto Y., Goto E., Higuchi A., Shimazaki J., Tsubota K. (2003). Albumin as a tear supplement in the treatment of severe dry eye. Br. J. Ophthalmol..

[25] Zhou Y.-J., Li L.-S., Sun J.-L., Guan K., Wei J.-F. (2019). 1H NMR-based metabolomic study of metabolic profiling for pollinosis. World Allergy Organ. J..

[26] Shine W.E., McCulley J.P. (1998). Keratoconjunctivitis Sicca Associated With Meibomian Secretion Polar Lipid Abnormality. Arch. Ophthalmol..

[27] Schuett B.S., Millar T.J. (2013). An investigation of the likely role of (O-acyl) ω-hydroxy fatty acids in meibomian lipid films using (O-oleyl) ω-hydroxy palmitic acid as a model. Exp. Eye Res..

[28] Sanchez V., Galor A., Jensen K., Mondal K., Mandal N. (2022). Relationships between ocular surface sphingomyelinases, Meibum and Tear Sphingolipids, and clinical parameters of meibomian gland dysfunction. Ocul. Surf..

[29] Zhang B., Hu S., Baskin E., Patt A., Siddiqui J.K., Mathé E.A. (2018). RaMP: A Comprehensive Relational Database of Metabolomics Pathways for Pathway Enrichment Analysis of Genes and Metabolites. Metabolites.

[30] Kovaničová Z., Karhánek M., Kurdiová T., Baláž M., Wolfrum C., Ukropcová B., Ukropec J. (2021). Metabolomic Analysis Reveals Changes in Plasma Metabolites in Response to Acute Cold Stress and Their Relationships to Metabolic Health in Cold-Acclimatized Humans. Metabolites.

[31] Braisted J., Patt A., Tindall C., Sheils T., Neyra J., Spencer K., Eicher T., A Mathé E. (2022). RaMP-DB 2.0: A renovated knowledgebase for deriving biological and chemical insight from metabolites, proteins, and genes. Bioinformatics.

[32] Peterson R.C., Wolffsohn J.S. (2009). Objective Grading of The Anterior Eye. Optom. Vis. Sci..

[33] Leonardi A., Lazzarini D., Valerio A.L.G., Scalora T., Fregona I. (2018). Corneal staining patterns in vernal keratoconjunctivitis: The new VKC-CLEK scoring scale. Br. J. Ophthalmol..

[34] Brown S.H.J., Kunnen C.M.E., Duchoslav E., Dolla N.K., Kelso M.J., Papas E.B., de la Jara P.L., Willcox M.D.P., Blanksby S.J., Mitchell T.W. (2013). A Comparison of Patient Matched Meibum and Tear Lipidomes. Investig. Opthalmol. Vis. Sci..

[35] Albietz J.M. (2000). Prevalence of Dry Eye Subtypes in Clinical Optometry Practice. Optom. Vis. Sci..

