# Supplementary material for: The Plight of the Metabolite: Oxidative Stress and Tear Film Destabilisation Evident in Ocular Allergy Sufferers across Seasons in Victoria, Australia
Source: Int J Mol Sci. 2024 Apr 4;25(7):4019. doi: 10.3390/ijms25074019 (PMC11012581; doi:10.3390/ijms25074019)
Supplement: Supplementary file 1 [file ijms-25-04019-s001.zip › ijms-2915107-SI.pdf]

Supplementary data Table S1. Differentially expressed metabolites between peak allergy season OA suffers (PA) and peak allergy season healthy controls (PHC), as well as off-peak season allergy sufferers (OPA) and off-peak season healthy controls (OPHC). All metabolites had a minimum Log2 fold-change of +/- 1.5, VIP values of > 1 and a p-value≤0.05 as determined by two sample Welch's t-tests. p≤0.05 (*), p≤0.02 (**), p≤0.01 (***). OAHFA- (O-acyl)-ω-hydroxy fatty acids; CE- cholesteryl ester; UK-CE- Unknown Cholesteryl Ester species; WE- wax ester; DE-I-Ch- type I diester; DE-II-Ch- type II diester; PC- phosphatidylcholine; PE- phosphatidylethanolamine; SM- sphingomyelin; TAG- triglyceride; DG- diglyceride.

Metabolite	Fold-change (log_2)	p-value	Significance
	Upregulated in PA/PHC		
Uracil	1.4955	0.036819	*
Hydroxylamine	1.6479	0.035163	*
Cytosine	1.6985	0.0434	*
Decanoic acid	1.7407	0.048926	*
Inositol	1.7568	0.0345	*
Nonanoic acid	1.9231	0.032138	*
Ethanolamine	1.974	0.0092015	***
Lauric acid	2.0131	0.048793	*
TAG(53:6)	2.0658	0.047593	*
Salicylic acid	2.1065	0.017881	**
Threonic acid	2.1291	0.013232	**
Trehalose	2.1393	0.026083	*
Maltose	2.1442	0.022889	**
Niacinamide	2.1481	0.032698	*
1-Butylamine	2.3559	0.025628	*
Octanoic acid	2.5657	0.039637	*
PC(37:2)	2.6215	0.023596	**
Methylsuccinic acid	2.9707	0.01414	**
Glycerol-3	3.0148	0.029682	*
Vanilmandelic acid	3.3264	0.013312	**
OAHFA(54:0)	3.4301	0.010141	***
DG(O-4:0_22:5)	3.6116	0.004867	***
Mannose	4.1316	0.049107	*
CE(27:1)	4.3654	0.010844	***
Gluconolactone	4.3837	0.0092689	***
L-cystine	6.206	0.044313	*
N-Acetyl-L-phenylalanine	8.312	0.038897	*
	Downregulated in PA/PHC		
Homogentisic acid	-5.6202	0.043959	*
Hexanoylglycine	-3.2619	0.0065767	***
Ornithine	-2.1103	0.04118	*
	Upregulated in OPA/OPHC		
Theanine	1.5046	0.0018488	***
Azelaic acid	1.5376	0.0013216	***
PC(38:2)	1.5441	0.008078	***
UK_CE_10	1.5787	0.04625	*
OAHFA(42:3)	1.5808	0.020133	**
Glutaric acid	1.6744	0.013312	**

PC(36:1)	1.6764	0.0070447	***
Niacinamide	1.6799	0.01088	***
DE-II 62:3	1.7048	0.031197	*
UK_CE_3	1.7627	0.023141	**
DE-I-Ch 46:2	1.7704	0.037372	*
UK_CE_6	1.7931	0.033845	*
DE-I-Ch 47:2	1.799	0.049614	*
WE(43:3)	1.8145	0.049257	*
DE-II 61:2	1.8333	0.019969	**
OAHFA(50:2)	1.8478	0.049241	*
TAG(53:2)	1.8579	0.048521	*
UK_CE_1	1.8658	0.019265	**
DE-II 64:4	1.8711	0.030463	*
DE-II 64:3	1.8724	0.035023	*
DE-II 65:3	1.8978	0.044752	*
DE-I-Ch 47:1	1.9027	0.031635	*
UK_CE_2	1.9208	0.031835	*
DE-I-Ch 48:2	1.921	0.035331	*
UK_CE_4	1.946	0.017753	**
UK_CE_5	1.9499	0.028134	*
DE-II 66:3	1.9539	0.027635	*
OAHFA(43:1)	1.9661	0.04122	*
DE-II 65:2	1.9741	0.03398	*
DE-II 63:2	1.987	0.034024	*
DE-I-Ch 49:1	1.9877	0.039036	*
DE-II 67:2	1.9877	0.022305	**
DE-II 63:3	1.9992	0.048414	*
UK_CE_7	2.0095	0.029848	*
DE-II 68:3	2.0171	0.03513	*
DE-I-Ch 49:2	2.0177	0.038548	*
DE-I-Ch 48:3	2.0226	0.032033	*
DE-II 67:3	2.0296	0.037614	*
OAHFA(44:1)	2.0368	0.030323	*
Adipic acid	2.0476	0.0030951	***
DE-I-Ch 39:0	2.0716	0.029774	*
DE-II 70:3	2.0724	0.03616	*
UK_CE_8	2.0794	0.029112	*
DE-I-Ch 51:5	2.0847	0.028944	*
DE-I-Ch 41:0	2.0877	0.034568	*
DE-II 66:4	2.1248	0.030055	*
DE-II 61:1	2.1256	0.026886	*
UK_CE_11	2.1548	0.02623	*
DE-I-Ch 51:2	2.1566	0.030702	*
DE-II 68:4	2.1602	0.023696	**
UK_CE_9	2.169	0.025139	*
DE-II 65:4	2.181	0.027699	*
DE-I-Ch 48:4	2.1832	0.031139	*
DE-I-Ch 50:2	2.1932	0.031621	*
DE-I-Ch 50:3	2.232	0.025833	*
OAHFA(48:2)	2.2489	0.0047937	***
DE-II 70:4	2.2801	0.022069	**

DE-I-Ch 52:2	2.3109	0.025681	*
DE-I-Ch 50:4	2.3178	0.023281	**
WE(46:3)	2.3202	0.047255	*
TAG(56:3)	2.4293	0.049261	*
DE-I-Ch 42:1	2.4313	0.013356	***
DE-I-Ch 42:2	2.4543	0.015065	**
DE-I-Ch 52:3	2.5261	0.016647	**
DE-I-Ch 52:4	2.5746	0.026534	*
DE-II 60:1	2.6865	0.026016	*
CE(24:1)	2.7886	0.046622	*
DE-I-Ch 52:5	2.849	0.0079534	***
DE-I-Ch 41:1	3.0272	0.020328	**
OAHFA(43:3)	3.1617	0.021948	**
OAHFA(45:2)	3.2016	0.04288	*
OAHFA(44:2)	3.3366	0.0010566	***
WE(48:3)	3.3383	0.039155	*
OAHFA(40:2)	3.3975	0.0079689	***
CE(25:0)	3.477	0.030941	*
OAHFA(49:2)	3.4902	0.0041888	***
OAHFA(46:2)	3.5717	0.00035948	***
Uridine	4.0366	0.048082	*
OAHFA(47:2)	4.0949	0.042489	*
Acetoacetic acid	4.5341	0.013569	***
OAHFA(51:2)	4.946	0.0068987	***
Tagatose	5.1446	0.014546	***
OAHFA(50:6)	5.5589	0.015442	**
	Downregulated in OPA/OPHC		
Pyridoxine	-1.927	0.039064	*
Sorbose	-1.6814	0.02022	**
Catechol	-1.5591	0.020238	**

Supplementary data Table S2. Differentially expressed metabolites among allergy sufferers and healthy controls across peak allergy season and off-peak season. All metabolites had a minimum Log2 fold-change of +/- 1.5, VIP values of > 1 and a p-value≤0.05 as determined by two sample Welch's t-tests. p≤0.05 (*), p≤0.02 (**), p≤0.01 (***). OAHFA- (O-acyl)-ω-hydroxy fatty acids; CE- cholesteryl ester; WE- wax ester; DE-I-Ch- type I diester; DE-II-Ch- type II diester; PC-phosphatidylcholine; PE-phosphatidylethanolamine; SM- sphingomyelin; TAG-triglyceride; DG- diglyceride.

Metabolite	Fold-change (log_2)	p-value	Significance
	Upregulated in PA/OPA		
OAHFA(46:0)	1.8219	0.033131	*
Epinephrine-3	2.3765	0.049868	*
Fructose 1-phosphate-meto-6	2.6012	0.022147	**
Psicose-meto-5	3.0132	0.027601	*
Pinitol-5	3.2739	0.038442	*
DG(O-4:0_22:5)	3.5137	0.005348	***
Glycerol-3	3.613	0.015592	**
Sorbose-meto-5.1	3.6943	0.0498	*
Xylulose-meto-4	4.6378	0.030692	*

5-Dehydroquinic acid-meto-4	5.6065	0.032653	*
	Downregulated in PA/OPA		
PE(18:2/22:6)	-1.9319	0.040596	*
	Upregulated in PHC/OPHC		
Adipic acid	1.5104	0.012405	***
OAHFA(42:1)	1.5652	0.036103	*
Phenylalanine	1.6039	0.021474	**
Leucine	1.7169	0.025477	*
OAHFA(44:1)	1.8774	0.024632	**
OAHFA(43:1)	1.8844	0.025759	*
Methionine	1.8984	0.0099615	***
Tyrosine	1.9555	0.021424	**
OAHFA(50:3)	1.983	0.048843	*
OAHFA(43:2)	2.0143	0.016793	**
OAHFA(44:0)	2.0709	0.025958	*
DE-I-Ch 42:2	2.1363	0.031688	*
DE-II 62:1	2.1515	0.046381	*
DG(16:1_18:2)	2.1833	0.028811	*
OAHFA(43:4)	2.2995	0.012623	***
WE(44:3)	2.484	0.032582	*
OAHFA(47:1)	2.5946	0.049298	*
WE(43:3)	2.6383	0.03371	*
Guanine	2.9757	0.021587	**
OAHFA(41:0)	2.9931	0.049998	*
WE(46:3)	3.0245	0.033224	*
OAHFA(44:2)	3.0509	0.02983	*
OAHFA(52:2)	3.3568	0.04955	*
DE-I-Ch 41:1	3.4408	0.029428	*
OAHFA(46:2)	3.5822	0.028274	*
Inosine	4.1857	0.04009	*
3-Hydroxyisovaleric acid	4.1982	0.047835	*
Uridine	4.2268	0.013148	***
WE(45:3)	4.2703	0.0082001	***
OAHFA(45:2)	5.7169	0.045157	*
OAHFA(51:2)	6.2113	0.0091643	***
	Downregulated in PHC/OPHC		
Eicosapentaenoic acid	-2.4758	0.0029644	***
SM(d35:2)	-2.3577	0.0071879	***
PC(34:4)	-2.3023	0.018937	**
PC(37:3)	-2.226	0.017761	**
PC(35:3)	-2.1655	0.02431	*
PE(18:0/20:3)	-2.1655	0.02431	*
Orotic acid	-1.5883	0.033866	*
